# Hypertension in women: the role of adolescent childbearing

**DOI:** 10.1186/s12889-021-11488-z

**Published:** 2021-07-29

**Authors:** Biplab K. Datta, Muhammad J. Husain, Deliana Kostova

**Affiliations:** 1 Global Noncommunicable Diseases Branch, Division of Global Health Protection, Center for Global Health, Centers for Disease Control and Prevention, 1600 Clifton Road, Atlanta, 30329 GA USA; 2 Present Address: Institute of Public and Preventive Health, Augusta University, 1120 15th Street, Augusta, 30912 GA USA

**Keywords:** Adolescent childbearing, Teenage pregnancy, Hypertension, Life course

## Abstract

**Background:**

Adolescent childbearing is associated with various health risks to the mother and child, and potentially with adverse socioeconomic outcomes. However, little is known about the role of adolescent childbearing in maternal health outcomes in adulthood. This study investigates the link between childbirth in adolescence and later-life risk of hypertension among women in India.

**Methods:**

We obtained nationally representative data on demographic and health outcomes for 442,845 women aged 25 to 49 from the India National Family Health Survey (NFHS) 2015-16. We assessed the difference in hypertension prevalence between women who gave birth in adolescence (age 10 to 19) and those who did not, for the full sample and various sub-samples, using linear probability models with controls for individual characteristics, hypertension risk factors, and geographic fixed effects.

**Results:**

Nearly 40% of the women in the sample gave birth in adolescence. The adjusted probability of being hypertensive in adulthood was 2.3 percentage points higher for this group compared to women who did not give childbirth in adolescence. This added probability was larger for women who gave birth earlier in adolescence (4.8 percentage points) and for women who gave birth more than once in adolescence (3.4 percentage points).

**Conclusions:**

Adolescent childbearing was strongly associated with a higher probability of adult female hypertension in India. This finding illustrates the intertemporal relationship between health risk factors during the life cycle, informing the importance of addressing adverse early life events (e.g. child marriage and adolescent childbirth) for hypertension outcomes among women in India.

## Background

Adolescent childbearing is associated with various health risks for the mother and child, and with adverse socioeconomic outcomes [1]. Adverse maternal and perinatal outcomes include eclampsia, puerperal endometritis, and systemic infections, while infants born to adolescent mothers suffer increased rates of severe neonatal complications, low birth weight and premature delivery [1–4]. Adolescent childbirth is disproportionately represented in socially vulnerable groups and can perpetuate the cycle of poverty. Its consequences on later life outcomes include reduced education opportunities, employment and income [5–7], and it has been linked to negative health behaviors such as smoking and drinking [8, 9], and child health and child education outcomes [7, 10].

The evidence on the effects of adolescent childbearing on mothers’ later-life physical health status is less extensive. Several epidemiological studies have documented links between the timing of fertility and conditions such as women’s mental health and depression [11, 12], post-reproductive mortality [13], diabetes [14], and limitations in activities of daily living [15]. Childbirth at an earlier age has been associated with higher risk of hypertension among women in Australia [16], Sweden [17] and South Korea [18]. However, the effects of early childbirth on hypertension in lower-income countries, where child marriage is considerably more common [19], are less well understood. Low-income countries face a growing hypertension burden and bear the brunt of premature cardiovascular diseases (CVD) mortality globally [20]. Evaluating the linkages between early childbirth and later-life hypertension in low-income areas can increase understanding of the connections between health events at different stages of the life cycle, informing the potential benefits of early intervention in reducing long-term health disparities.

This study analyzes the relationship between adolescent childbirth and later-life hypertension among women of reproductive age in India. Both early childbirth and hypertension are critical public health issues in India, where hypertension is the top underlying cause of disease burden [21]. Although the prevalence of hypertension in men (27%) is higher than that in women (20%) [22], nearly two thirds of excess female deaths at age 45 to 69 in India have been attributed to CVD [23]. Social determinants of hypertension, including human and social development, have a significant explanatory role in hypertension in India [22]. In women, added exposure to circulating estrogen and metabolic effects resulting from early childbirth may contribute to later cardiovascular complications [18], which can also be aggravated by higher birth parity [24]. As an adverse socioeconomic and physiological factor that disproportionately affects women [1], adolescent childbirth has the potential to exacerbate broad health disadvantages among women in India.

With the world’s largest adolescent population, India is one of the top three countries that account for 30% of all adolescent childbirths globally [25]. Adolescent childbearing in India has implications for pregnancy outcomes, child nutrition, access to antenatal or postnatal care, and mother’s reproductive health [26–28], but has not been explored in relation to women’s hypertension outcomes. By investigating the relationship between early childbearing and later risk of hypertension in India, we document a new perspective on the possible mechanisms that may help shape chronic health outcomes in vulnerable populations.

## Methods

### Data

We obtained data on 442,845 women aged 25 to 49 from the India National Family Health Survey (NFHS–4) 2015-16. The NFHS–4 is part of the USAID’s Demographic and Health Survey (DHS) program. The DHS survey protocols are reviewed and approved by the ICF Institutional Review Board (IRB). Details of the ethical review are available at: https://dhsprogram.com/Methodology/Protecting-the-Privacy-of-DHS-Survey-Respondents.cfm.

NFHS–4 provides respondent’s age at first birth, from which we derive variables indicating whether the individual gave birth during adolescence (age 10 to 19). We confined the sample to age 25+ since most (44%) of the women in the survey, aged 20 to 49, gave first childbirth during age 20 to 24. NFHS-4 also provides measures of respondent’s systolic (SBP) and diastolic blood pressure (DBP), and documents self-reported anti-hypertensive medication intake. An individual was defined as hypertensive if SBP ≥ 140*mmHg* and/or DBP ≥ 90*mmHg* and/or the individual reported taking anti-hypertensive medication at the time of the survey [29].

### Empirical analysis

We analyzed the differences in hypertension prevalence between women aged 25-49 who gave birth between the ages of 10 to 19 and those who did not. We estimated the following linear probability model:

1 $$\begin{array}{@{}rcl@{}} HTN_{i} &= \beta_{0} + \beta_{1} Adolescent childbirth_{i} + \mathbf{X}_{i}\mathbf{\beta_{3}} + \mathbf{Risk}_{i} \mathbf{\beta_{4}}  \\ & + \beta_{5} Urban_{i} + Agegroup + State + \epsilon_{i}. \end{array} $$

Where *HTN*_*i*_ is a binary variable equal to 1 if individual i has hypertension, and zero otherwise; *Adolescentchildbirth*_*i*_ is a binary variable equal to 1 if individual *i* gave birth between the ages of 10 to 19, and zero otherwise; **X**_*i*_ is a vector of individual characteristics including body mass index (BMI) status in four categories (underweight: < 18.5 *kg*/*m*^2^, normal: 18.5−24.9 *kg*/*m*^2^, overweight: 25.0−29.9 *kg*/*m*^2^, or obese: ≥ 30.0 *kg*/*m*^2^), wealth index quintiles, level of education (none, primary, secondary, and higher), marital status (not married, married, formerly married), religion (Hindu, Muslim, Christian, Sikh, Buddhist, other), and caste (distinguishing between women who are part of officially designated socially disadvantaged ‘backward’ groups, termed ‘scheduled caste’, ‘scheduled tribe’, ‘other backward class’, and women who are not). **Risk**_*i*_ is a vector of five individual hypertension risk factors: tobacco or alcohol use, oral contraceptive use, lifetime parity (number of births), menopause status and current pregnancy status. *Urban*_*i*_ is a binary variable that takes the value of 1 if individual i resides in an urban area and zero if rural area.

*Agegroup* denotes age group fixed effects for the five age categories (25 to 29, 30 to 34, 35 to 39, 40 to 44, and 45 to 49). *State* denotes the state fixed effects and *ε*_*i*_ is idiosyncratic error term. State fixed effects capture the state level variations in resources and policies (e.g., access to female education, healthcare facilities) that influence our variables of interest. Complex survey weights were used to obtain regression estimates. We applied the regression model to the full sample and to subgroups of women by 5-year age groups. The statistical analysis was conducted using Stata 13.1 software. The coefficient of interest, *β*_1_, shows the adjusted difference in the probability of being hypertensive between women who gave birth in adolescence and those who did not. We also estimated a binary logistic specification, and the marginal effects were found very similar to those of the linear probability model.

Next, we explored expanded versions of the model in Equation 1 by expanding the indicator Adolescent Childbirth into subcategories according to type of birth. The expanded version follows the following specification:

2 $$\begin{array}{@{}rcl@{}} HTN_{i}\! &= \!\alpha_{0} \,+\, \sum\limits_{j=1}^{J-1} \gamma_{j} Adolescentbirthtype_{ji} \,+\, \mathbf{X}_{i}\mathbf{\alpha_{3}} \!+ \mathbf{\!Risk}_{i} \mathbf{\alpha_{4}}  \\ & + \alpha_{5} Urban_{i} + Agegroup \!+ State + \mu_{i} \end{array} $$

Where *Adolescentbirthtype*_*ji*_ represents the subcategory of each adolescent childbirth across *j* subcategories, and Eq. 2 was estimated using separate regressions that explore four different childbirth scenarios related to: 1) the stage of adolescence in which childbirth occurs, 2) the number of adolescent childbirths, 3) adolescent pregnancy outcome (childbirth vs. termination), and 4) adolescent childbirth in the context of child marriage.

Childbirth can occur at different stages of adolescence. We defined ‘early’ adolescent childbearing as giving birth at age 15 or younger [30], ‘middle’ adolescent childbearing as giving birth at age 16 to 17, and ‘late’ adolescent childbearing as giving birth at age 18 to 19 [31]. To explore differences based on the stage of adolescence in which childbirth can occur, we estimated Eq. 2 where the *j* number of categories represented by the indicator *Adolescentbirthtype*_*ji*_ denote mutually-exclusive stages of the time of childbirth for individual *i*: early, middle and late adolescence, with ‘no adolescent childbirth’ as the base category. In case of multiple adolescent childbirths at different stages of adolescence, the time of the first childbirth was used to determine the appropriate stage.

Next, we explored differences associated with the number of births in adolescence. This was done by estimating a version of Eq. 2 where the categories represented by the indicator *Adolescentbirthtype*_*ji*_ denote the occurrence of single or multiple adolescent births, with ‘no adolescent childbirth’ as the base category.

The NFHS–4 provides information on whether a pregnancy was ever terminated, and at what age such event occurred. Based on this information, we defined four pregnancy subcategories: no pregnancy in adolescence, childbirth and no terminated pregnancy in adolescence, no childbirth but terminated pregnancy in adolescence, and both terminated pregnancy and childbirth in adolescence. We estimated a version of Equation 2 where the indicators denoted as *Adolescentbirthtype*_*ji*_ represent this set of categories, with ‘no adolescent pregnancy’ as the base category.

Lastly, we examined differences related to marriage at the time of birth. Child marriage in India is defined as being married before the legal age of 18 (for female). We estimated Eq. 2 where the indicators denoted by *Adolescentbirthtype*_*ji*_ represent the following set of categories: did not get married before age 18 and did not give birth in adolescence (base category), did not get married before age 18 but gave birth in adolescence, was married before age 18 but did not give birth in adolescence, and was married before age 18 and gave birth in adolescence.

#### Subsample analysis

The estimated association between adolescent childbirth and later hypertension can be confounded if it reflects bias from unobserved characteristics that may simultaneously predispose women to adolescent childbearing while also raising the risk of hypertension in later life, for example through health behavior and lifestyle. To address such bias, in addition to controlling for a appropriate set of covariates, we explored the sensitivity of the results to restricting the analysis to subsamples of women with shared select risk characteristics, mitigating within-group selection bias.

Teenage pregnancy in India is disproportionately higher in rural areas, among women with low or no education, among women at lower wealth quintiles, and among women who belong to certain castes [29]. We estimated the Kaplan-Meier survival functions (adjusted for age groups) for the event of giving first childbirth for subgroups of women living in rural and urban areas, women with primary or no education and higher than primary education, women at the lowest two wealth quintiles and at the highest wealth quintile, and women belonging to designated educationally and socially disadvantaged groups including scheduled castes scheduled tribes and other. We performed a log-rank test to compare the survival distributions across the subgroups and found that the survival probability of childbearing at age 19 significantly differs across subgroups. Thus, to check the robustness of our baseline results against possible selection bias based on cross-group differences, we estimated Equation 1 for the eight different socioeconomic risk subgroups.

Behavioral factors such as tobacco and alcohol use are common risk factors for many CVD [32, 33]. Because these behaviors can be related to factors that may also determine early reproductive behavior, we conducted the analysis on the subgroup of women who have ever consumed tobacco or alcohol. Since other factors such as healthcare utilization can be similarly related to both hypertension status and reproductive behavior, we conducted the analysis on subgroups of women who had previously received a hypertension measurement and women who had not. We further checked the robustness of our results for subgroups based on birth parity: women having 1-2 children, 3-4 children, 5+ children; and on subgroups of women who have reached menopause and women currently using oral contraceptives.

## Results

Table 1 presents summary means of adolescent childbearing and hypertension among reproductive-age women in India. Approximately 40% of the women aged 25 to 49 in India gave birth in adolescence. Around half of the adolescent childbirths occurred in late adolescence and a third occurred in middle adolescence. As expected, hypertension prevalence was the lowest for the youngest age group and increased with age. Across all age groups, hypertension prevalence was higher for women who gave birth in adolescence than for women who did not. Table 2 provides background characteristics of the study participants.

**Table 1:** Summary statistics of adolescent childbearing and hypertension in women aged 25-49, India NFHS 2015-16

	Childbirth in adolescence (%)				Hypertension	Diff. in
	Overall	Early:	Middle:	Late:	Prevalence	Prevalence
		*Age*≤15	*Age*16−17	*Age*18−19		
					(%)	(% points)
All ages	39.51	7.00	12.65	19.85	15.53	2.66***
	(39.24, 39.77)	(6.88, 7.13)	(12.49, 12.82)	(19.66, 20.04)	(15.35, 15.71)	(2.33, 3.00)
Age 25 to 29	34.11	5.05	10.44	18.63	7.25	1.09***
	(33.67, 34.56)	(4.85, 5.25)	(10.15, 10.72)	(18.29, 18.98)	(7.01, 7.48)	(0.60, 1.58)
Age 30 to 34	40.56	6.77	13.26	20.53	11.22	2.14***
	(40.04, 41.09)	(6.51, 7.02)	(12.93, 13.60)	(20.13, 20.93)	(10.92, 11.53)	(1.50, 2.78)
Age 35 to 39	42.07	7.9	13.54	20.62	15.98	2.16***
	(41.53, 42.60)	(7.61, 8.20)	(13.17, 13.91)	(20.21, 21.03)	(15.61, 16.34)	(1.43, 2.88)
Age 40 to 44	43.27	8.71	14.16	20.41	21.61	2.04***
	(42.71, 43.83)	(8.39, 9.02)	(13.75, 14.57)	(19.98, 20.83)	(21.16, 22.05)	(1.13, 2.95)
Age 45 to 49	39.39	7.45	12.62	19.32	27.02	3.36***
	(38.84, 39.95)	(7.16, 7.75)	(12.26, 12.99)	(18.87, 19.76)	(26.51, 27.52)	(2.33, 4.38)

Estimates are obtained using complex survey weights. 95% confidence intervals are in parenthesis.

Hypertension is defined as SBP ≥ 140*mmHg* and/or DBP ≥ 90*mmHg* and/or taking anti-hypertensive medication.

Difference in prevalence refers to unadjusted difference in hypertension prevalence between women with and without adolescent childbirth experience.

∗∗∗*p*<0.01,∗∗*p*<0.05,∗*p*<0.1

**Table 2:** Background characteristics of women aged 25-49, India NFHS 2015-16

	All ages	25 to 29	30 to 34	35 to 39	40 to 44	45 to 49
Nutritional status						
Normal (BMI 18.5 – 24.9)	0.565	0.611	0.576	0.56	0.529	0.523
	(0.562, 0.567)	(0.607, 0.616)	(0.571, 0.581)	(0.555, 0.566)	(0.523, 0.534)	(0.517, 0.528)
Underweight (BMI <18.5)	0.161	0.204	0.166	0.144	0.14	0.133
	(0.160, 0.163)	(0.201, 0.208)	(0.163, 0.170)	(0.141, 0.147)	(0.137, 0.144)	(0.129, 0.136)
Overweight (BMI 25.0-29.9)	0.204	0.146	0.197	0.218	0.24	0.249
	(0.202, 0.206)	(0.143, 0.150)	(0.192, 0.201)	(0.213, 0.222)	(0.235, 0.245)	(0.244, 0.254)
Obese (BMI ≥30.0)	0.07	0.038	0.061	0.078	0.091	0.095
	(0.068, 0.071)	(0.036, 0.040)	(0.059, 0.064)	(0.075, 0.081)	(0.088, 0.095)	(0.092, 0.099)
Wealth index quintile						
Quintile 1 (Poorest)	0.176	0.176	0.186	0.179	0.17	0.161
	(0.173, 0.178)	(0.173, 0.180)	(0.183, 0.190)	(0.176, 0.183)	(0.166, 0.174)	(0.157, 0.165)
Quintile 2 (Poorer)	0.189	0.188	0.188	0.192	0.189	0.186
	(0.186, 0.191)	(0.184, 0.192)	(0.184, 0.192)	(0.188, 0.196)	(0.185, 0.193)	(0.182, 0.190)
Quintile 3 (Middle)	0.202	0.207	0.198	0.201	0.198	0.202
	(0.199, 0.204)	(0.203, 0.212)	(0.194, 0.202)	(0.197, 0.205)	(0.193, 0.202)	(0.198, 0.207)
Quintile 4 (Richer)	0.214	0.214	0.215	0.212	0.214	0.212
	(0.211, 0.216)	(0.210, 0.219)	(0.210, 0.219)	(0.207, 0.217)	(0.209, 0.219)	(0.207, 0.218)
Quintile 5 (Richest)	0.221	0.214	0.213	0.216	0.229	0.238
	(0.217, 0.225)	(0.209, 0.219)	(0.207, 0.219)	(0.210, 0.221)	(0.223, 0.235)	(0.232, 0.244)
Education						
No education	0.368	0.228	0.311	0.389	0.46	0.537
	(0.365, 0.371)	(0.224, 0.233)	(0.306, 0.315)	(0.384, 0.394)	(0.454, 0.466)	(0.530, 0.543)
Primary	0.145	0.135	0.143	0.154	0.15	0.15
	(0.144, 0.147)	(0.132, 0.138)	(0.139, 0.146)	(0.150, 0.158)	(0.146, 0.154)	(0.146, 0.154)
Secondary	0.38	0.462	0.429	0.371	0.322	0.264
	(0.377, 0.383)	(0.457, 0.467)	(0.423, 0.434)	(0.365, 0.376)	(0.316, 0.328)	(0.259, 0.270)
Higher	0.106	0.175	0.118	0.087	0.069	0.049
	(0.104, 0.109)	(0.170, 0.179)	(0.114, 0.122)	(0.083, 0.090)	(0.065, 0.072)	(0.046, 0.052)
Marital status						
Never in union	0.032	0.083	0.023	0.013	0.01	0.008
	(0.031, 0.032)	(0.080, 0.086)	(0.022, 0.025)	(0.011, 0.014)	(0.009, 0.011)	(0.007, 0.009)
Married	0.908	0.895	0.941	0.927	0.901	0.868
	(0.906, 0.909)	(0.892, 0.898)	(0.938, 0.943)	(0.924, 0.930)	(0.898, 0.904)	(0.864, 0.871)
Widowed, divorced or separated	0.061	0.022	0.036	0.06	0.089	0.124
	(0.060, 0.062)	(0.021, 0.023)	(0.034, 0.038)	(0.057, 0.063)	(0.086, 0.092)	(0.120, 0.128)
Religion						
Hindu	0.813	0.8	0.814	0.814	0.82	0.824
	(0.809, 0.818)	(0.795, 0.806)	(0.809, 0.820)	(0.808, 0.820)	(0.815, 0.826)	(0.818, 0.829)
Muslim	0.127	0.139	0.127	0.128	0.119	0.113
	(0.123, 0.131)	(0.134, 0.144)	(0.123, 0.132)	(0.122, 0.133)	(0.114, 0.124)	(0.108, 0.117)
Christian	0.026	0.024	0.024	0.026	0.026	0.03
	(0.024, 0.027)	(0.022, 0.026)	(0.022, 0.026)	(0.024, 0.027)	(0.024, 0.028)	(0.028, 0.032)
Sikh	0.018	0.018	0.018	0.017	0.019	0.018
	(0.017, 0.019)	(0.017, 0.019)	(0.017, 0.019)	(0.016, 0.019)	(0.018, 0.020)	(0.017, 0.019)
Buddhist	0.01	0.01	0.009	0.009	0.01	0.009
	(0.009, 0.011)	(0.009, 0.012)	(0.008, 0.011)	(0.008, 0.011)	(0.008, 0.011)	(0.008, 0.011)
Other	0.007	0.008	0.007	0.006	0.006	0.006
	(0.006, 0.008)	(0.007, 0.009)	(0.006, 0.008)	(0.005, 0.007)	(0.005, 0.007)	(0.005, 0.007)
Caste						
None	0.275	0.264	0.275	0.276	0.285	0.282
	(0.271, 0.280)	(0.259, 0.270)	(0.270, 0.281)	(0.270, 0.282)	(0.279, 0.292)	(0.275, 0.288)
Scheduled caste	0.2	0.207	0.203	0.198	0.193	0.196
	(0.196, 0.204)	(0.202, 0.212)	(0.197, 0.209)	(0.193, 0.203)	(0.188, 0.199)	(0.190, 0.201)
Scheduled tribe	0.09	0.095	0.091	0.088	0.086	0.088
	(0.088, 0.092)	(0.092, 0.099)	(0.088, 0.094)	(0.085, 0.091)	(0.083, 0.089)	(0.084, 0.091)
Other backward class	0.434	0.433	0.43	0.438	0.435	0.435
	(0.430, 0.438)	(0.427, 0.439)	(0.424, 0.436)	(0.432, 0.444)	(0.429, 0.441)	(0.429, 0.442)
Lifetime parity						
None	0.076	0.167	0.064	0.042	0.035	0.035
	(0.075, 0.077)	(0.163, 0.170)	(0.061, 0.066)	(0.040, 0.044)	(0.033, 0.038)	(0.033, 0.037)
1-2	0.468	0.59	0.515	0.447	0.383	0.334
	(0.465, 0.471)	(0.585, 0.595)	(0.510, 0.520)	(0.442, 0.453)	(0.377, 0.389)	(0.328, 0.340)
3-4	0.341	0.224	0.347	0.383	0.399	0.405
	(0.339, 0.344)	(0.221, 0.228)	(0.342, 0.352)	(0.378, 0.388)	(0.393, 0.404)	(0.399, 0.411)
5+	0.114	0.019	0.074	0.128	0.183	0.226
	(0.113, 0.116)	(0.018, 0.020)	(0.072, 0.076)	(0.125, 0.131)	(0.179, 0.187)	(0.221, 0.231)
Tobacco/alcohol use	0.101	0.06	0.082	0.106	0.13	0.155
	(0.099, 0.103)	(0.057, 0.062)	(0.079, 0.084)	(0.103, 0.109)	(0.127, 0.134)	(0.151, 0.159)
Menopause	0.058	0	0.006	0.019	0.079	0.238
	(0.056, 0.059)	(0.000, 0.000)	(0.006, 0.007)	(0.017, 0.020)	(0.076, 0.082)	(0.233, 0.244)
Oral contraceptive use	0.035	0.049	0.048	0.036	0.021	0.009
	(0.034, 0.036)	(0.046, 0.051)	(0.045, 0.050)	(0.034, 0.038)	(0.020, 0.023)	(0.008, 0.011)
Currently pregnant	0.03	0.081	0.031	0.01	0.002	0.001
	(0.029, 0.030)	(0.079, 0.084)	(0.030, 0.033)	(0.009, 0.011)	(0.002, 0.003)	(0.001, 0.001)
Urban	0.354	0.349	0.353	0.357	0.363	0.351
	(0.351, 0.358)	(0.343, 0.355)	(0.347, 0.359)	(0.352, 0.363)	(0.357, 0.368)	(0.345, 0.357)
Observations	442,845	112,813	95,073	88,668	75,091	71,200

95% confidence intervals are in parenthesis.

Estimates are obtained using complex survey weights

**Fig. 1:**
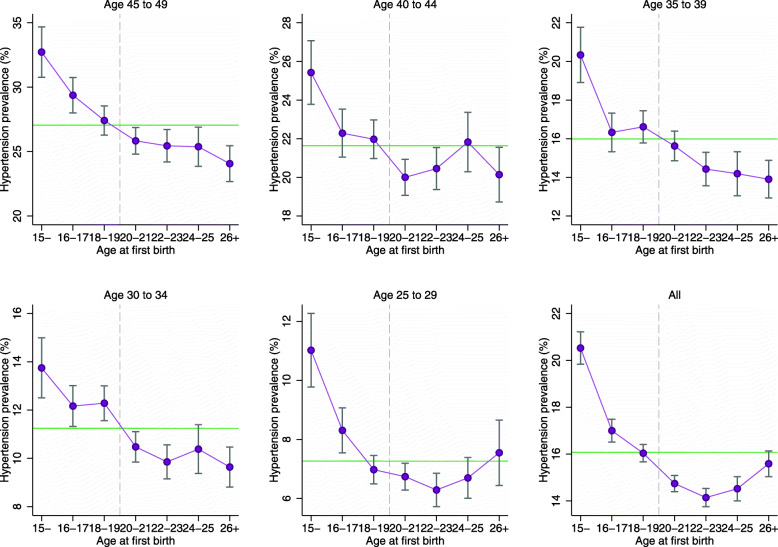
Hypertension prevalence by age group and age at first birth in women aged 25-49, India NFHS 2015-16. Estimates were obtained using complex survey weights. Vertical lines across the markers represent 95% confidence intervals. Vertical axis labels are different for different age groups because of different hypertension prevalence levels across age groups. The horizontal line indicates the average hypertension prevalence of the respective age groups. First birth at 15 includes women who gave birth between age 10 to 15

Figure 1 depicts the relationship between age at first birth and hypertension prevalence. For all examined age groups, the potential risk of having hypertension is higher for women who gave birth in early adolescence. Except for the 25-to-29 age group, hypertension prevalence is also higher for women who gave birth in middle and late adolescence than those who gave first birth in later periods. Since nearly two-thirds of the currently pregnant women in the sample belongs to the 25-to-29 age group, the relatively higher hypertension prevalence for women in this age group may be related to pregnancy induced hypertension.

The unadjusted trends reported in Table 1 and Fig. 1 motivate further investigation of the role of adolescent childbirth in later-life hypertension. Regression-adjusted estimates of the difference in the probability of being hypertensive are shown in Table 3, where the hypertension probability estimate was 2.3 percentage points (pp) higher for women who gave birth in adolescence. These findings were consistent across all age groups, with adjusted differences in the probability of having hypertension ranging from 0.8pp for the youngest age group to 3.2pp for the oldest age group. Other covariates that were strongly associated with raising the probability of being hypertensive were overweight and obese status, menopause or currently using oral contraceptives, being ever married, belonging to a religious minority group (Muslim, Christian, Sikh, or other), belonging to a scheduled tribe, and tobacco or alcohol use among younger women.

**Table 3:** Estimates from linear regression models of the probability of being hypertensive among women aged 25-49, India NFHS 2015-16

	All ages	25 to 29	30 to 34	35 to 39	40 to 44	45 to 49
Adolescent childbirth	0.023***	0.008***	0.024***	0.023***	0.024***	0.032***
	(0.020, 0.027)	(0.002, 0.013)	(0.017, 0.031)	(0.015, 0.031)	(0.015, 0.034)	(0.022, 0.043)
Nutritional status^a^						
Underweight (BMI <18.5)	-0.033***	-0.014***	-0.020***	-0.041***	-0.059***	-0.066***
	(-0.036, -0.029)	(-0.019, -0.009)	(-0.027, -0.014)	(-0.049, -0.033)	(-0.068, -0.049)	(-0.077, -0.054)
Overweight (BMI 25.0-29.9)	0.088***	0.046***	0.068***	0.088***	0.118***	0.127***
	(0.084, 0.093)	(0.038, 0.054)	(0.059, 0.077)	(0.078, 0.098)	(0.106, 0.130)	(0.114, 0.139)
Obese (BMI ≥30.0)	0.178***	0.089***	0.142***	0.183***	0.214***	0.234***
	(0.169, 0.187)	(0.072, 0.106)	(0.123, 0.160)	(0.164, 0.201)	(0.194, 0.234)	(0.214, 0.254)
Wealth index quintile^b^						
Quintile 2 (Poorer)	-0.006***	-0.007*	-0.008*	-0.004	-0.004	-0.002
	(-0.011, -0.002)	(-0.014, 0.000)	(-0.016, 0.001)	(-0.014, 0.007)	(-0.017, 0.008)	(-0.016, 0.012)
Quintile 3 (Middle)	-0.006**	-0.011***	-0.007	0.001	-0.002	0.004
	(-0.011, -0.000)	(-0.019, -0.004)	(-0.017, 0.003)	(-0.011, 0.012)	(-0.015, 0.012)	(-0.011, 0.020)
Quintile 4 (Richer)	0.005*	-0.008*	0.005	0.013*	0.012	0.018*
	(-0.001, 0.011)	(-0.017, 0.000)	(-0.006, 0.016)	(-0.001, 0.027)	(-0.004, 0.028)	(-0.000, 0.036)
Quintile 5 (Richest)	-0.005	-0.010*	-0.005	0.003	-0.01	-0.002
	(-0.012, 0.003)	(-0.021, 0.000)	(-0.018, 0.009)	(-0.014, 0.019)	(-0.029, 0.009)	(-0.023, 0.019)
Education^c^						
Primary	0.006**	0.001	0.003	-0.007	0.006	0.012
	(0.001, 0.011)	(-0.006, 0.009)	(-0.006, 0.013)	(-0.018, 0.004)	(-0.008, 0.019)	(-0.004, 0.027)
Secondary	-0.002	-0.007**	-0.005	-0.010**	0.001	0.018**
	(-0.006, 0.003)	(-0.013, -0.001)	(-0.014, 0.003)	(-0.020, -0.000)	(-0.012, 0.014)	(0.003, 0.033)
Higher	-0.020***	-0.015***	-0.024***	-0.036***	-0.018	-0.012
	(-0.028, -0.013)	(-0.025, -0.005)	(-0.038, -0.010)	(-0.054, -0.018)	(-0.041, 0.004)	(-0.043, 0.020)
Marital status^d^						
Married	0.012**	0.004	0.002	0.026	0.031	0.026
	(0.002, 0.023)	(-0.008, 0.016)	(-0.024, 0.027)	(-0.011, 0.063)	(-0.019, 0.081)	(-0.034, 0.085)
Widowed, divorced, separated	0.011*	-0.008	0.013	0.024	0.029	0.025
	(-0.001, 0.024)	(-0.025, 0.010)	(-0.016, 0.041)	(-0.016, 0.064)	(-0.022, 0.081)	(-0.035, 0.086)
Religion^e^						
Muslim	0.024***	0.012***	0.016***	0.032***	0.039***	0.033***
	(0.018, 0.030)	(0.004, 0.019)	(0.006, 0.026)	(0.019, 0.045)	(0.023, 0.055)	(0.015, 0.051)
Christian	0.017***	0.005	0.025*	-0.001	0.028	0.031*
	(0.004, 0.030)	(-0.014, 0.025)	(-0.004, 0.054)	(-0.025, 0.023)	(-0.009, 0.065)	(-0.004, 0.066)
Sikh	0.017**	0.020*	0.011	0.015	0.023	0.014
	(0.003, 0.030)	(-0.001, 0.040)	(-0.015, 0.038)	(-0.013, 0.044)	(-0.014, 0.059)	(-0.023, 0.051)
Buddhist	0.002	-0.015	0.015	0.033	-0.004	-0.02
	(-0.022, 0.025)	(-0.036, 0.007)	(-0.024, 0.054)	(-0.035, 0.102)	(-0.062, 0.054)	(-0.090, 0.051)
Other	0.023**	0.004	0.069**	-0.02	0.045	0.02
	(0.000, 0.045)	(-0.019, 0.028)	(0.007, 0.130)	(-0.056, 0.016)	(-0.028, 0.118)	(-0.040, 0.079)
Caste^f^						
Scheduled caste	0.001	0.002	-0.008	0.005	0.005	0.008
	(-0.004, 0.007)	(-0.005, 0.010)	(-0.018, 0.002)	(-0.007, 0.018)	(-0.010, 0.020)	(-0.008, 0.023)
Scheduled tribe	0.014***	0.006	0.007	0.01	0.016*	0.042***
	(0.008, 0.021)	(-0.003, 0.016)	(-0.004, 0.019)	(-0.005, 0.024)	(-0.002, 0.034)	(0.023, 0.062)
Other backward class	-0.001	0	-0.002	-0.002	-0.002	0
	(-0.006, 0.003)	(-0.006, 0.007)	(-0.010, 0.006)	(-0.012, 0.009)	(-0.015, 0.010)	(-0.013, 0.014)
Urban	-0.003	-0.004	-0.007*	-0.007	-0.002	0.004
	(-0.008, 0.002)	(-0.010, 0.002)	(-0.015, 0.000)	(-0.016, 0.003)	(-0.014, 0.010)	(-0.009, 0.017)
Lifetime parity^g^						
1-2	-0.030***	-0.015***	-0.019**	-0.055***	-0.045***	-0.071***
	(-0.038, -0.023)	(-0.024, -0.006)	(-0.035, -0.003)	(-0.079, -0.032)	(-0.074, -0.017)	(-0.102, -0.040)
3-4	-0.036***	-0.014***	-0.029***	-0.058***	-0.064***	-0.078***
	(-0.045, -0.028)	(-0.024, -0.004)	(-0.046, -0.012)	(-0.081, -0.034)	(-0.092, -0.035)	(-0.109, -0.047)
5+	-0.048***	-0.014*	-0.032***	-0.062***	-0.063***	-0.087***
	(-0.057, -0.039)	(-0.031, 0.002)	(-0.052, -0.013)	(-0.087, -0.037)	(-0.093, -0.033)	(-0.119, -0.055)
Tobacco/alcohol use	0.002	0.014***	0.010*	0.003	-0.005	-0.001
	(-0.003, 0.007)	(0.004, 0.025)	(-0.000, 0.021)	(-0.008, 0.015)	(-0.017, 0.008)	(-0.014, 0.012)
Menopause	0.043***	-	0.068***	0.047***	0.054***	0.037***
	(0.034, 0.052)		(0.025, 0.112)	(0.021, 0.073)	(0.036, 0.072)	(0.025, 0.049)
Oral contraceptive use	0.016***	0.014**	0.020**	0.030***	0.030*	0.01
	(0.007, 0.026)	(0.001, 0.026)	(0.004, 0.036)	(0.009, 0.051)	(-0.004, 0.064)	(-0.040, 0.061)
Currently pregnant	-0.037***	-0.030***	-0.047***	-0.077***	-0.043	-0.066
	(-0.043, -0.031)	(-0.037, -0.023)	(-0.059, -0.034)	(-0.097, -0.056)	(-0.118, 0.031)	(-0.189, 0.057)
Observations	442,845	112,813	95,073	88,668	75,091	71,200
Age-group Fixed Effect	Yes	-	-	-	-	-
State Fixed Effect	Yes	Yes	Yes	Yes	Yes	Yes

∗∗∗*p*<0.01,∗∗*p*<0.05,∗*p*<0.1. 95% confidence intervals are in parenthesis.

Estimates represent the added probability of being hypertensive. Estimates were obtained using linear probability models (LPM) with complex survey weights.

^a^Relative to normal (BMI 18.5 – 24.9).

^b^Relative to Quintile 1 (Poorest).

^c^Relative to no education.

^d^Relative to never married.

^e^Relative to Hindu.

^f^Relative to not designated socially backward class.

^g^Relative to no childbirth

Table 4 presents estimates from the expanded models of hypertension and adolescent childbearing. Panel A reports estimates for childbearing at different stages of adolescence. The added probability of being hypertensive was the largest for women who gave birth in early adolescence (4.8pp) and the lowest for women who gave birth in late adolescence (1.6pp). The added probability of hypertension associated with prior childbirth in early adolescence ranged from 3.5pp for younger women to 6.4pp for the oldest women. Panel B shows a comparison between single and multiple adolescent childbirths. On average, the added probability of hypertension was larger for women who gave more than one childbirth in adolescence, whose risk of hypertension was twice as high as the added probability for women with a single adolescent childbirth and 3.4pp higher than women with no adolescent births. Panel C shows that experiencing a terminated pregnancy in adolescence is not associated with added probability of later-life hypertension while experiencing a childbirth raises the hypertension probability by 2.2pp. Panel D shows that child marriage alone was associated with higher probability of later-life hypertension, but the added probability was higher when the marriage is accompanied by birth in adolescence.

**Table 4:** Estimates from linear regression models of the probability of being hypertensive among women aged 25-49, India NFHS 2015-16, by adolescence stage of childbirth, adolescent childbirth parity, adolescent pregnancy status, and child marriage

	All ages	25 to 29	30 to 34	35 to 39	40 to 44	45 to 49
**Panel A**						
Adolescence stage of childbirth^a^						
Early (*Age*≤15)	0.048***	0.035***	0.036***	0.050***	0.051***	0.064***
	(0.041, 0.055)	(0.022, 0.048)	(0.023, 0.050)	(0.035, 0.065)	(0.034, 0.069)	(0.043, 0.085)
Middle (*Age*16−17)	0.022***	0.010**	0.022***	0.017***	0.019***	0.034***
	(0.016, 0.027)	(0.002, 0.019)	(0.013, 0.032)	(0.006, 0.028)	(0.005, 0.032)	(0.019, 0.050)
Late (*Age*18−19)	0.016***	0	0.022***	0.018***	0.018***	0.020***
	(0.012, 0.020)	(-0.006, 0.006)	(0.013, 0.030)	(0.008, 0.028)	(0.007, 0.030)	(0.007, 0.033)
Observations	442,845	112,813	95,073	88,668	75,091	71,200
**Panel B**						
Adolescent childbirth parity^b^						
Single	0.017***	0.003	0.022***	0.019***	0.016***	0.026***
	(0.013, 0.021)	(-0.003, 0.009)	(0.014, 0.030)	(0.009, 0.028)	(0.005, 0.027)	(0.014, 0.038)
Multiple	0.034***	0.020***	0.029***	0.031***	0.039***	0.044***
	(0.029, 0.039)	(0.011, 0.029)	(0.019, 0.038)	(0.020, 0.042)	(0.026, 0.051)	(0.029, 0.059)
Observations	442,845	112,813	95,073	88,668	75,091	71,200
**Panel C**						
Adolescent pregnancy status^c^						
Adolescent childbirth	0.022***	0.005*	0.023***	0.023***	0.023***	0.032***
	(0.018, 0.026)	(-0.000, 0.011)	(0.016, 0.030)	(0.015, 0.031)	(0.013, 0.032)	(0.021, 0.043)
Terminated pregnancy	0.009	-0.019**	0.016	0.026	0.028	-0.017
	(-0.008, 0.026)	(-0.036, -0.001)	(-0.018, 0.050)	(-0.015, 0.068)	(-0.029, 0.084)	(-0.072, 0.038)
Both	0.045***	0.033***	0.041***	0.033**	0.075***	0.043**
	(0.032, 0.057)	(0.010, 0.055)	(0.020, 0.062)	(0.005, 0.061)	(0.035, 0.116)	(0.000, 0.086)
Observations	442,722	112,792	95,039	88,640	75,070	71,181
**Panel D**						
Adolescent childbirth and						
child marriage^d^						
No marriage but childbirth	0.021***	0.000	0.018**	0.036***	0.029***	0.027**
	(0.014, 0.028)	(-0.011, 0.010)	(0.003, 0.033)	(0.018, 0.054)	(0.009, 0.050)	(0.004, 0.050)
Marriage but no childbirth	0.012***	0.005	0.021***	0.013**	0.009	0.014*
	(0.007, 0.018)	(-0.003, 0.014)	(0.011, 0.032)	(0.001, 0.025)	(-0.005, 0.024)	(-0.002, 0.030)
Marriage and childbirth	0.026***	0.010***	0.030***	0.022***	0.028***	0.037***
	(0.022, 0.030)	(0.004, 0.016)	(0.022, 0.037)	(0.013, 0.032)	(0.017, 0.039)	(0.025, 0.050)
Observations	413,769	109,590	91,021	82,938	68,157	62,063

∗∗∗*p*<0.01,∗∗*p*<0.05,∗*p*<0.1. 95% confidence intervals are in parenthesis.

Estimates represent the added probability of being hypertensive. Estimates were obtained using linear probability models (LPM) with complex survey weights. All models control for the following (not shown): state fixed effects, nutritional status, wealth index quintile, education, marital status, current pregnancy status, religion, caste, lifetime parity, menopause, tobacco or alcohol use, oral contraceptive use, urban/rural residence, and age group (all-age specification only). ^a^Relative to no birth in adolescence. ^b^Relative to no birth in adolescence. ^c^Relative to no pregnancy in adolescence. ^d^Relative to no child marriage and no birth in adolescence

**Fig. 2:**
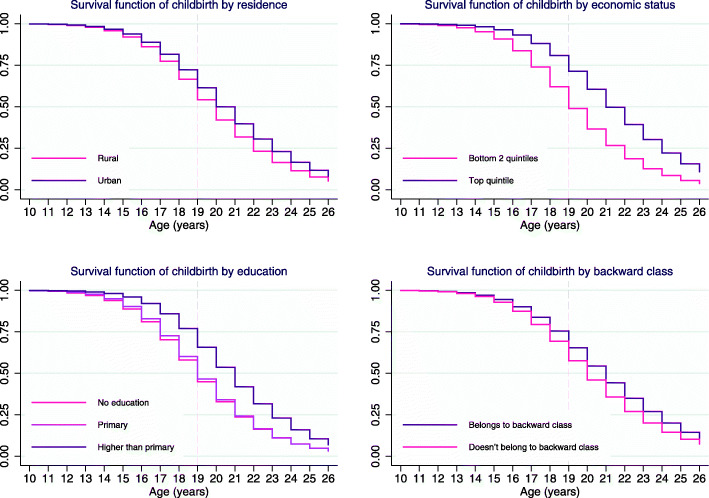
Kaplan-Meier survival analysis of childbirth, by socioeconomic status. Survival probabilities are adjusted for age groups (birth cohorts)

Figure 2 shows the Kaplan-Meier survival functions for the event of first childbirth. At age 19, the survival probability of childbearing (adjusted for age group) was 0.54 in rural areas and 0.61 in urban areas; 0.49 at the bottom two wealth quintiles and 0.71 at the top quintile; 0.45 to 0.47 for individuals with primary or no education and 0.66 for individuals with higher than primary education; and 0.55 for individuals of designated ‘backward’ social class and 0.61 for individuals not in this class. The log rank test for each set of groups also suggests that the survival distributions are different across the respective groups. Panel A in Table 5 shows the adjusted differences in the probability of being hypertensive between women who gave birth in adolescence and those who did not for the socioeconomic subgroups in the survival analysis. The estimates were robust across all subgroups. The result was robust for the subgroups of women in the bottom two wealth quintiles (added probability of hypertension, 1.9pp), women of designated ‘backward’ social class (added probability of hypertension, 2.3pp), women with primary or no education (added probability of hypertension, 2.4pp), and women in rural areas (added probability of hypertension, 2.3pp).

**Table 5:** Estimates from linear regression models of the probability of being hypertensive among women aged 25-49, India NFHS 2015-16, by hypertension risk subgroup

**Panel A**				
(Socioeconomic risk groups)				
	Residence		Wealth quintile	
	Urban	Rural	Bottom 2	Top 1
Adolescent childbirth	0.028***	0.023***	0.019***	0.034***
	(0.021, 0.036)	(0.019, 0.027)	(0.014, 0.023)	(0.023, 0.044)
Observations	132,228	310,617	175,391	87,567
	Education		Backward class	
	No/ Primary	Higher	Yes	No
Adolescent childbirth	0.024***	0.023***	0.023***	0.023***
	(0.019, 0.028)	(0.017, 0.028)	(0.019, 0.027)	(0.015, 0.031)
Observations	231,783	211,062	329,810	113,035
**Panel B**				
(Clinical risk groups)				
	Parity			Menopause
	Children 1-2	Children 3-4	Children 5+	
Adolescent childbirth	0.019***	0.028***	0.018***	0.028***
	(0.013, 0.025)	(0.022, 0.033)	(0.010, 0.027)	(0.011, 0.046)
Observations	189,161	157,525	58,041	25,927
	OCP	Tobacco/Alcohol	Prior hypertension screening	
			Yes	No
Adolescent childbirth	0.016	0.021***	0.025***	0.019***
	(-0.003, 0.034)	(0.012, 0.031)	(0.020, 0.029)	(0.014, 0.025)
Observations	17,147	68,073	315,043	127,787

∗∗∗*p*<0.01,∗∗*p*<0.05,∗*p*<0.1. 95% confidence intervals are in parenthesis.

Estimates represent the added probability of being hypertensive in women with prior adolescent childbirth relative to women with no adolescent childbirth. Estimates were obtained using linear probability models (LPM) with complex survey weights. All models control for the following (not shown unless indicated): state fixed effects, nutritional status, wealth index quintile, education, marital status, current pregnancy status, religion, caste, lifetime parity, menopause, tobacco or alcohol use, oral contraceptive use, urban/rural residence, and age group fixed effect

In the data, we found that women (age 30+) at menopause are 10.8 percentage points more likely to have hypertension. Similarly, oral contraceptive user women are 0.8 percentage points more likely to have hypertension. We also found parity being associated with increasing risk of hypertension as women with 1 to 2 children are 1.5 percentage points more likely to have hypertension, while women with 3 to 4 children and 5+ children are respectively 3.6 and 5.1 percentage points more likely to have hypertension than women without any children. Lastly, we found that tobacco or alcohol consuming women are 2.4 percentage points more likely to have hypertension in the NFHS–4 data.

Panel B in Table 5 shows the adjusted differences in probability of being hypertensive across risk factor subgroups such as menopause, contraceptive use, parity, and tobacco/alcohol use. Across all risk subgroups, the added probability of being hypertensive was higher for women who gave birth during adolescent age compared to those who did not. The results were also consistent for the group of women who previously had their blood pressure screened.

## Discussion

We find that women who gave birth in adolescence have a higher probability of being hypertensive in adulthood. The added probability of hypertension is highest for those who gave birth in early adolescence, decreasing for childbirth events in later stages of adolescence. The findings were robust across age groups and various socioeconomic, demographic, and hypertension-risk sub-groups.

Our estimates indicate that the added risk of later-life hypertension associated with adolescent childbirth is higher for women who have had multiple adolescent births than for those with only one, suggesting that it may be cumulative. No added risk was detected for women with terminated adolescent pregnancies, suggesting that the added hypertension risk may be related to the physiological demands of carrying a pregnancy. We further estimate that the added probability of later-life hypertension is magnified when the adolescent childbirth occurs within the context of a child marriage, and that child marriage is independently associated with higher risk of hypertension in later life. These findings suggest that the socioeconomic disadvantages from child marriage can enhance the biological mechanisms through which early childbirth may affect later-life hypertension.

Applying the analysis to subgroups defined by factors that independently affect both hypertension and reproductive outcomes can help to address concerns about selection bias in the baseline estimates. We find that the added risk of later-life hypertension associated with adolescent childbirth is statistically robust across higher-risk groups such as women who use tobacco/alcohol, use contraception, have high lifetime parity, and have lower wealth, education or healthcare use. Although our estimates do not inform about the precise mechanism through which early birth might determine subsequent hypertension in women, and the estimates cannot be interpreted as causal due to remaining confounding factors that may play a role, our findings are consistent with the hypothesis that early reproductive activity may increase subsequent cardiovascular risk among women in India. This is consistent with findings from high-income countries, where similar correlations have been documented for women in Australia [16], Sweden [17] and South Korea [18].

Our findings have important implications in the Indian context. Vascular diseases linked to hypertension are one of the top mortality risks for women aged 15 to 69 in India [34]. Access to adequate hypertension care and management is limited, and there are large variations in the hypertension care cascade across regions [35]. In our sample of reproductive-age women, nearly three out of four hypertension cases were untreated, raising the risk of ischemic heart disease [36], the leading cause of deaths in India [37]. Gender discrimination in access to healthcare in India [38] further aggravates women’s health risks linked to hypertensive conditions. With limited or no access to health insurance, the out-of-pocket spending on hypertension treatment can lead to catastrophic level of healthcare spending [39] and can adversely impact household resource allocation [40]. Despite high need for hypertension care, studies have shown critical gaps in the ability to deliver hypertension management services in primary care outlets in India [41, 42]. The added risk of hypertension among women who gave birth in adolescence, combined with the high frequency of child marriage and adolescent births in India, can contribute to straining future health system resources. Since child marriage is more common in rural areas and in vulnerable population, the added hypertension risk from adolescent childbirth can exacerbate existing health disparities.

As all observational studies, the present analysis is subject to estimation limitations that prevent the identification of causal effects. Because it uses self-reported survey data, some statistics may be subject to recall bias. The hypertensive status in the survey was not clinically diagnosed, rather determined based on blood pressure measurement during one occasion and self-reported anti-hypertensive medication intake. Further, it does not inform about the precise clinical mechanism that might explain the link between adolescent childbirth and later-life hypertension. However, by documenting the significant and robust association between early birth and subsequent hypertension status, it motivates further investigation of the biological and social consequence of early reproductive behavior.

## Conclusions

The literature on adolescent childbearing has evolved mostly around direct adverse outcomes such as complications of pregnancy and childbirth leading to maternal mortality, perinatal and infant mortality, and children’s health. Sociological research has described its role in reducing opportunities for future education and employment, increasing social stigma, intimate partner violence, and perpetuating the poverty cycle [1]. We expand the existing evidence by documenting the link between adolescent childbearing and later-life hypertension in women in India, where efforts to reduce child marriage are ongoing. Despite progress in preventing child marriage, a large number of women in India still got married before the age of 18 [43] and most of these women gave birth during adolescence [29]. We show that the probability of adult female hypertension in India is disproportionately higher among these women who experienced adolescent childbirth.

Analyzing health issues from a life course perspective offers a holistic understanding of the problem and facilitates early interventions that could reduce the burden of health problems in later life. Our findings illustrate the relevance of early reproductive health to long-term health outcomes of women in the low-and-middle-income countries.

## Data Availability

The datasets used and/or analysed during the current study are freely available from the USAID’s DHS Program website (https://www.dhsprogram.com/data/dataset_admin/login_main.cfm) upon registering as a DHS data user and submitting a research proposal.

## Abbreviations

CVD: Cardiovascular disease
NFHS–4: India national family health survey round 4
DHS: Demographic and health survey
SBP: Systolic blood pressure
DBP: Diastolic blood pressure
BMI: Body mass index
OCP: Oral contraceptive pill
